# Turmeric as a Possible Treatment for COVID-19-Induced Anosmia and Ageusia

**DOI:** 10.7759/cureus.17829

**Published:** 2021-09-08

**Authors:** A. Bert Chabot, Margaret P Huntwork

**Affiliations:** 1 Department of Surgery, Tulane University School of Medicine, New Orleans, USA; 2 Department of Clinical Immunology, Allergy & Rheumatology, Tulane University School of Medicine, New Orleans, USA

**Keywords:** curcumin, covid-19, coronavirus, sars-cov-2, anosmia, ageusia, hyposmia, dysgeusia, turmeric

## Abstract

The coronavirus disease 2019 (COVID-19) pandemic has caused a calamitous perturbation of society worldwide. Anosmia and ageusia (or hyposmia and dysgeusia) have been recognized as two common expressions of COVID-19 infection that linger for days to weeks, and in rare cases are thought to be immutable. Time alone has hitherto proven most effective in their restoration, but turmeric, long believed by some to have medicinal properties, may be a swifter and surer alleviator of anosmia and ageusia. This case series reports the speedy and consequential restoration of taste and smell in two subjects infected with COVID-19 following ingestion of one 1000 mg dose of a turmeric supplement. In view of this, we propose that turmeric be considered in the treatment of anosmia and ageusia caused by COVID-19 infection.

## Introduction

Anosmia and ageusia are common and often comorbid symptoms of coronavirus disease 2019 (COVID-19). The frequency of olfactory and gustatory dysfunction in infected individuals has been reported to be 19.4-88% [1]. While most of those infected recover smell and taste, up to 20% experience persistent anosmia or hyposmia and ageusia or dysgeusia after 30 days [2].

The mechanisms by which COVID-19 causes anosmia and ageusia are unknown; they may be similar to those of other coronaviruses. Upper respiratory viruses are known to cause nasal mucosal swelling and olfactory cleft conductive blockade [3]. There is also evidence that severe acute respiratory syndrome coronavirus 2 (SARS-CoV-2) takes advantage of the proximity of epithelial and neuronal cells in olfactory mucosa, and the virus crosses the neuronal-mucosal interface just behind the cribriform plate [4]. Infection of supporting cells and vascular pericytes belonging to the olfactory epithelium and bulb has been theorized to hamper olfactory neuron function [5]. Possible stem-cell involvement may also explain residual or permanent anosmia.

The mechanism of COVID-19-induced ageusia may relate to the angiotensin-converting enzyme 2 (ACE2) cellular receptor, which is widely expressed in mucous membranes of the oral cavity, modulates taste perception, and is a SARS-CoV-2 binding receptor [1]. SARS-CoV-2 also binds to sialic acid receptors [1]. Sialic acid delays the enzymatic degradation of glycoproteins that transport gustatory molecules in taste pores [1]. SARS-CoV-2 may increase the gustatory threshold by binding sialic acid receptors on the taste buds, hastening the destruction of gustatory particles [1]. It may also be that ageusia is attendant on anosmia, due to the close functional relationship these sensory systems share.

Current treatment for individuals without spontaneous recovery of smell within two weeks of COVID-19 infection includes olfactory training, a safe and inexpensive approach that has shown some promise in the alleviation of postinfectious anosmia [6]. This requires scents such as lemon, rose, clove, and eucalyptus to be sniffed for 20 seconds each, no less than twice daily, for three months or longer [6]. Corticosteroids are not recommended, as they have not been sufficiently proven to be effective in the treatment of postinfectious anosmia, and in fact, may be harmful [6]. Intranasal sodium citrate, intranasal vitamin A, and systemic omega-3 may possibly be of benefit in postinfectious anosmia; however, like olfactory training and corticosteroids, they have not been proven to be efficacious in COVID-19-induced anosmia [6]. While recovery of smell may aid in that of taste, to the best of our knowledge, no specific treatment has been recommended for recovery of taste alone.

We present two individuals who experienced an improvement in taste and smell after taking turmeric supplements.

## Case presentation

Subject one is a 25-year-old man with no chronic medical condition, prior nasal or oral conditions, or recognized risk factors for anosmia or ageusia, who developed acute onset headache, chest tightness, lower extremity and extraocular myalgias, fever, and fatigue on day one. All symptoms were rated as mild in intensity. Symptoms were associated with total ageusia and anosmia, with taste and smell self-reported as 1/10 in intensity, where one is no taste or smell whatsoever, and ten is normal, unmitigated taste and smell. While most symptoms resolved on day five, anosmia and ageusia persisted without improvement for 46 days. He tested positive for COVID-19 via polymerase chain reaction (PCR) testing on day 15. Oral aspirin, diphenhydramine, prednisone, and famotidine taken early in the course of infection provided no relief. On day 46, the subject ingested a capsule supplement containing 1000 mg of turmeric extract (95% curcuminoids) and 10 mg of black pepper extract. He experienced complete restoration of smell and taste, with both senses rated 10/10, ten minutes after supplement ingestion.

Subject two is a 28-year-old man with mild intermittent asthma, but no other prior nasal or oral conditions, or recognized risk factors for anosmia or ageusia, who developed acute onset of chest tightness, fever, headache, nausea, and knee arthralgias, rated as mild in intensity, on day one. Three days later, he developed near-anosmia and ageusia with both senses, respectively, rated as 2/10 and 1/10 in intensity. On day one, he tested positive for COVID-19 via PCR testing. On day two, he started a daily regimen of azithromycin, vitamin D, vitamin C, quercetin, zinc, and dexamethasone. These medications failed to restore any measure of taste or smell. On day four, he took a single dose of a capsule supplement containing 1000 mg of turmeric extract (95% curcuminoids), 1000 mg of *Boswellia serrata* plant extract, and 15 mg of black pepper extract for the first time. Twelve hours after taking the turmeric supplement, the subject reported experiencing a restoration of both smell and taste to intensity of 6/10 each, with 10/10 being achieved three days later. His other symptoms of chest tightness, fever, headache, nausea, and arthralgia persisted unabated until day nine.

## Discussion

The two subjects described herein experienced marked improvement in their anosmia/hyposmia and ageusia minutes to hours after one-time ingestion of 1000 mg of turmeric. Curcumin, the main component of the spice turmeric, is derived from the rhizome of the plant *Curcuma longa* [7,8]. Beneficial effects of curcumin have been reported over centuries in the treatment of various ailments, from oncological to autoimmune disorders [7]. Curcumin has been observed to block pro-inflammatory regulators and signaling pathways, including inhibiting nuclear factor (NF)-kappa B activation and suppressing tumor necrosis factor-alpha (TNF-α), interleukin 1 beta (IL-1β), interleukin 6 (IL-6), monocyte chemotactic protein-1 (MCP-1), prostaglandin E2, and cyclooxygenase-II, among others [7]. Yet the anti-inflammatory properties of curcumin are not without controversy; systematic reviews and meta-analyses have not found sufficient evidence to suggest decreased inflammation after turmeric use [8]. Curcumin has also been shown to inhibit various cytochrome P450 enzymes, necessitating extra prudence for patients who take medications reliant on cytochrome P450 for metabolism [9].

It is worth considering a one-time dose of 1000 mg of turmeric to those experiencing anosmia or ageusia following COVID-19 infection. Turmeric supplements are commonly produced in capsule form with Bioperine, or black pepper extract, which increases curcumin absorption and bioavailability [7,8]. This combination is therefore proffered as the ideal formulation.

Several mechanisms by which turmeric may alleviate anosmia or ageusia induced by COVID-19 are plausible. Curcumin has been shown to bind and block the active site of Mpro, the main protease utilized by COVID-19 to produce proteins required for viral replication from viral genomic RNA [10]. Curcumin may also hinder the formation of the COVID-19 spike protein-ACE2 complex, preventing viral entry into cells [11]. The anti-inflammatory action of curcumin may reduce nasal mucosal swelling.

Limitations of our case report include the use of a subjective scale to describe the loss of taste and smell. There were no pre-turmeric or post-turmeric measurements of the presence or accuracy of taste and smell. While one person mentioning that they regained taste and smell after ingesting turmeric is an anecdote, two people reporting improvement post-turmeric is a potential pattern. Further investigation with an objective study design is warranted.

## Conclusions

In conclusion, we present two individuals who experienced significant improvement in taste and smell shortly after ingestion of one dose of a turmeric supplement. The risk of one dose of turmeric is low in healthy individuals not on medications metabolized by cytochromes P450, and the potential benefit of regaining senses of taste and smell is high. Additional objective studies are needed to determine whether treatment with turmeric is helpful across a broader population and if so, via what mechanism of action.
